# Protective Effects of Betaine on Boar Sperm Quality during Liquid Storage and Transport

**DOI:** 10.3390/ani14182711

**Published:** 2024-09-19

**Authors:** Chenxuan Li, Chenxi Liu, Yingqi Chen, Yuting Zhao, Meiling Tan, Bin He

**Affiliations:** 1 Key Laboratory of Animal Physiology & Biochemistry, Ministry of Agriculture and Rural Affairs, College of Veterinary Medicine, Nanjing Agricultural University, Nanjing 210095, China; 2022207005@stu.njau.edu.cn (C.L.); chenxiliu@stu.njau.edu.cn (C.L.); 13288386738@163.com (Y.C.); zhaoyuting12138@163.com (Y.Z.); 18473448821@163.com (M.T.); 2 MOE Joint International Research Laboratory of Animal Health & Food Safety, Nanjing Agricultural University, Nanjing 210095, China

**Keywords:** sperm, betaine, semen extender, shipping, boar

## Abstract

**Simple Summary:**

Liquid-preserved boar semen is widely used in the pig breeding industry. Preservation and transportation can affect the fertilizing ability of sperm, reducing the efficiency of artificial insemination. Betaine, a natural plant extract, has been shown to benefit male fertility. However, it remains unclear whether it exerts a protective effect on liquid boar sperm during preservation and transportation. The results showed that the addition of appropriate amounts of betaine to boar semen extenders effectively mitigated the adverse effects of road transport and room temperature storage on sperm quality. Specifically, betaine supplementation showed significant protective effects against temperature fluctuation and vibration stress that inevitably occurred during transportation. The increased antioxidant capacity of boar sperm may underlie its beneficial effects. These findings offer a theoretical foundation for the use of betaine in artificial insemination procedures.

**Abstract:**

Boar semen is commonly used in artificial insemination (AI) for pig breeding, but its quality can be negatively affected by liquid preservation and transportation, leading to reduced fertility rates. Vibration and temperature fluctuations are critical factors that significantly impact semen quality during storage and transportation, influencing the success rate of AI procedures. Betaine, a naturally occurring compound known for its role in maintaining male fertility, demonstrates potential for improving the preservation and transportation of liquid-preserved boar sperm. The present study demonstrated that betaine supplementation in the semen extenders at 0.5 mg/mL had a significant protective effect on boar sperm motility during storage at 17 °C for 3 to 5 days. During road transportation, 2.5 mg/mL betaine showed significant protective effects on boar sperm progressive motility, while 0.4 mg/mL betaine notably improved boar sperm mitochondrial activity and antioxidant capacity, and reduced lipid peroxidation damage. Simulation models also demonstrated that betaine supplementation increased the proportion of sperm displaying progressive motility and possessing intact acrosomes, regardless of the storage temperature (17 °C or 25 °C), and effectively mitigated the damage caused by vibration at a speed of 200 r/min. Overall, supplementing liquid-preserved boar semen extenders with betaine shows promise in mitigating damage to sperm quality during storage and transportation.

## 1. Introduction

Artificial insemination (AI) is the primary biotechnology used to enhance production efficiency in the global pig industry. With the advance of AI, centralization of boar studs has become necessary to produce extended semen doses with strict and specialized quality control [1]. While sperm cryopreservation offers the advantage of extended storage and facilitates international trade, the cooling and freeze-thaw processes can adversely impact boar sperm [2]. These processes can cause membrane instability, resulting in lipid disorder and compromised acrosomal integrity. Consequently, the fertilization capacity of boar sperm is significantly reduced [3,4,5]. Therefore, the transportation of preserved semen in its liquid state remains the primary form of AI utilized in pigs today.

To ensure the longevity and quality of the boar semen, it is commonly diluted with a commercial extender, cooled to 15–18 °C, and promptly transported from the AI center to the breeder [6]. After transportation to the sow farm, the semen will be preserved at this temperature until insemination [7]. Despite rigorous quality control during production, various exogenous factors can impact boar sperm function, including the temperature at which semen is diluted [8,9], procedures used [8], and storage conditions [8,10]. Moreover, challenges persist while transporting liquid-preserved semen, such as temperature fluctuation and vibration emission [11,12]. The impact of these factors is manifested in the subsequent long-term storage through decreased motility, mitochondrial activity, membrane integrity, and heat resistance [13]. Therefore, it is essential to incorporate active ingredients into semen extenders to mitigate the impact of adverse factors and maintain sperm function.

Betaine, a naturally occurring compound derived from the trimethyl derivative of glycine, is found in plants, animals, and microorganisms [14]. Betaine is extensively utilized as a feed additive in animal husbandry due to its well-established efficacy in enhancing antioxidant capacity and promoting growth performance. Moreover, betaine has been demonstrated to possess protective effects on sperm in multiple species [15,16,17]. Dietary betaine supplementation has been shown to effectively mitigate the decline in sperm quality and fertility caused by heat stress in roosters [17]. Additionally, supplementing the cryopreservation solution for mouse sperm with 1% betaine was found to protect against mitochondrial cryopreservation damage, thereby preserving sperm motility [15]. However, these studies primarily focused on the cryoprotective properties of betaine and its potential role in enhancing sperm motility in cryopreserved semen or resistance to heat stress damage [18]. Limited research has been conducted on the impact of betaine supplementation on the storage and transportation of liquid-preserved boar sperm collected at room temperature.

In this study, both actual highway transportation models and simulation models are used to investigate the impact of temperature fluctuations and vibration stress on the quality of boar sperm during semen transportation. Our specific focus was to determine whether adding betaine to the diluted boar sperm could mitigate these adverse effects. The main objective of this study was to provide a theoretical framework for pig production practices and the advancement of AI technology.

## 2. Materials and Methods

### 2.1. Semen Collection and Processing

Mature and healthy Duroc boars were housed in individual pens with straw bedding and provided with a nutritionally balanced standard diet. Ejaculates without the gel fraction were collected using the gloved-hand technique at a local AI center. The sperm concentration ranged from 2.7 × 10^8^ to 31.1 × 10^8^ sperm per mL. The gross motility of the sperm ranged between 90.5% and 96.7%. The compositions and added amounts of the Modena solution, according to Chen et al. [19], are presented in Table 1. Betaine was purchased from Sigma-Aldrich (≥98.0%, W422312, St. Louis, MO, USA). Initially, 1 g of betaine was dissolved in 400 mL of sperm diluent to create a 2.5 mg/mL betaine solution. Subsequently, 1 and 0.4 mg/mL betaine solutions were prepared through further dilution.

### 2.2. Experimental Design

The experiment consisted of four parts.

Experiment 1 was designed to evaluate whether betaine offered protection to boar sperm stored at room temperature (17 °C). Following collection, the fresh boar semen was evenly distributed into four separate groups. To these groups, betaine was added at varying concentrations, 0, 0.5, 2.5, and 12.5 mg/mL, to the diluent containing boar sperm. The samples were then stored at 17 °C. The sperm motility was tracked on days 1, 3, and 5.

Experiments 2–4 were designed to investigate the potential protective impact of betaine on boar sperm during transportation. In Experiment 2, betaine was added at varying concentrations (0, 0.4, 1, and 2.5 mg/mL) to the diluent containing boar sperm. This was followed by the addition of these diluted samples to freshly collected boar semen by the boar station personnel. The semen samples were transported to the laboratory via express delivery, covering a distance of 370 km in approximately 16 h. Upon arrival at the laboratory, the semen samples were stored in a pre-cleaned and disinfected biochemical incubator maintained at a temperature of 17 °C. The samples were gently inverted and shaken twice daily to prevent sperm deposition. The sperm quality-related indicators were evaluated.

In Experiments 3 and 4, the semen collection procedure was identical to that of Experiment 2. Semen samples were assigned to a control group or a betaine-supplemented group with a concentration of 0.4 mg/mL. In Experiment 3, semen samples were incubated in biochemical incubators at various temperatures (11, 17, and 25 °C) for either 24 or 72 h to mimic temperature fluctuations during transportation, while betaine was added to the diluent containing boar sperm. In Experiment 4, semen samples were exposed to a thermostatic oscillator operating at either 0 or 200 rpm for 4 h to simulate bumps during transportation, while betaine was added to the diluent containing boar sperm [20]. After the treatments, sperm quality-related indicators were evaluated.

### 2.3. Sperm Motility Analysis

Sperm motility was evaluated by a computer-assisted sperm motility analyzer (Sperm Vision^®^ v. 3.5; Minitube of America, Verona, WI, USA) as previously described [21]. Briefly, a 5 μL droplet of preheated diluted semen was placed in a 20 μm deep disposable counting chamber that stayed in the MiniTherm^®^ stage warmer (Hamilton Thorne Inc., Beverly, MA, USA) at 37 °C during the analysis. Four randomly selected fields were measured 5 times each, obtaining 20 scans from which the average was used for the statistical analysis. The analysis was repeated 1 min later on the same counting chamber which remained on the warming plate at 37 °C throughout the interval. The total motility (%) and progressive motility (%) were assessed at 17 °C for 3 or 7 d, or 37 °C for 0, 3, or 6 h.

### 2.4. Evaluation of Mitochondrial Membrane Potential (ΔΨm)

Sperm ΔΨm were measured using a JC-1 fluorescent probe. Sperm at 10 × 10^6^ cells/mL were incubated at 37 °C for 3 h or at 17 °C for 24 h before analysis by flow cytometry (FACS Verse™, BD Biosciences, San Jose, CA, USA) according to previous publications [22,23]. Briefly, treated sperm were centrifuged at 1000× *g* for 5 min and then stained with 2.5 μg/mL JC-1 for 15 min at 37 °C. Following a wash with ice-cold PBS two times, samples were analyzed by flow cytometry at a flow rate of 10,000 events per second per sample. Fluorescence intensity was measured for J-aggregates (red fluorescence) at 535 nm excitation and 595 nm emission wavelength, and JC-1 monomers (green fluorescence) excitation and emission wavelength were at 485/535 nm, respectively. Sperm with high ΔΨm form JC-1 aggregates and fluoresce red; those with low ΔΨm contain monomeric JC-1 and fluoresce green.

### 2.5. Evaluation of Acrosomal Integrity

Coomassie brilliant blue G250 staining was used for acrosomal integrity analysis [19]. Sperm were washed twice in PBS by brief centrifugation at 300× *g* for 2 min and fixed in 3.7% paraformaldehyde/PBS for 30 min. The samples were suspended and spread on slides for air drying. The sperm smear was stained in 0.25% Coomassie Blue G250 for 2 min and washed in H_2_O. The air-dried slides were mounted and then checked for the percentage of acrosome intact sperm in at least 200 cells. The acrosome-intact sperm were stained purple-blue, and the posterior part of the acrosome was either not stained or stained a light purple.

### 2.6. Measurement of Antioxidant Capacity

The total antioxidant capacity (T-AOC) test kit from Nanjing Jiancheng Bioengineering Research Institute (A015-1-2, Nanjing, China) was used following the instructions. The semen samples were sonicated after adding precooled PBS, then centrifuged at 4 °C (12,000 r/min, 5 min), and the supernatant was aspirated for further use. The protein concentration of samples was tested using the BCA protein quantitative assay kit (P0012S, Beyotime Biotechnology Ltd., Shanghai, China). After mixing the samples with the working solution and application solution according to the instructions, the samples were incubated at room temperature for 6 min. The absorbance was measured at a wavelength of 402 nm using a microplate reader, and the corresponding concentration of total antioxidant capacity of semen samples was calculated in combination with the measured protein concentration.

### 2.7. Lipid Peroxidation Level Detection

The colorimetric lipid oxidation (MDA) assay kit (S0131S, Beyotime Biotechnology Ltd., Shanghai, China) was used for the determination of lipid peroxidation in semen samples by the instructions. After ultrasonic fragmentation, the semen samples were centrifuged at 4 °C (10,000–12,000× *g* for 10 min), and the supernatant was used for subsequent determination. The protein concentration of samples was tested using the BCA protein assay kit (P0012S, Beyotime Biotechnology Ltd., Shanghai, China). The reaction system was freshly prepared as requested, then run in a boiling water bath for 15 min. After the water bath, the samples were immediately cooled to room temperature on ice. After centrifugation at 1000× *g* for 10 min at room temperature, 200 μL of the supernatant was added to a 96-well plate, and the absorbance was subsequently measured at 532 nm using a microplate reader. The MDA content of semen samples was calculated according to the absorbance and standard curve.

### 2.8. Statistical Analysis

All data were tested for normality and variance homogeneity before statistical analysis. A paired-sample *t*-test was performed to assess the differences in sperm motility, ΔΨm, and acrosomal integrity. Two-way ANOVA was performed to assess the main effects of betaine and temperature or vibration, as well as their interactions on sperm motility and acrosomal integrity using the GLM, followed by the least significant difference post-hoc analysis to evaluate differences between specific groups. All analyses were performed using SPSS 20.0 software (IBMCorp., Armonk, NY, USA). Data are expressed as mean ± SEM. Two-tailed *p* values < 0.05 were considered statistically significant.

## 3. Results

### 3.1. Effects of Betaine Supplementation on Boar Sperm Motility Stored at Room Temperature (17 °C)

On the first day of storage, the lower concentrations of betaine at 0.5 and 2.5 mg/mL did not exhibit significant beneficial effects. However, after 3 days of storage at 17 °C, the supplementation of 0.5 mg/mL and 2.5 mg/mL betaine resulted in significantly improved total and progressive motility compared to the control group (*p* = 0.0447, *p* = 0.0257, respectively, Table 2). Following a 5-day storage period at 17 °C, the supplementation of 0.5 mg/mL betaine led to a notable enhancement in sperm progressive motility (*p* = 0.0272, Table 2). It is worth noting that throughout the entire 5-day storage period, the supplementation of betaine at a concentration of 12.5 mg/mL had a profoundly inhibitory impact on total and progressive motility on days 0, 1, 3, and 5 (*p* < 0.0001, *p* < 0.0001, *p* < 0.0001, respectively, Table 2).

### 3.2. Effects of Betaine Supplementation on Boar Sperm Quality during Semen Transportation

**Figure 1:**
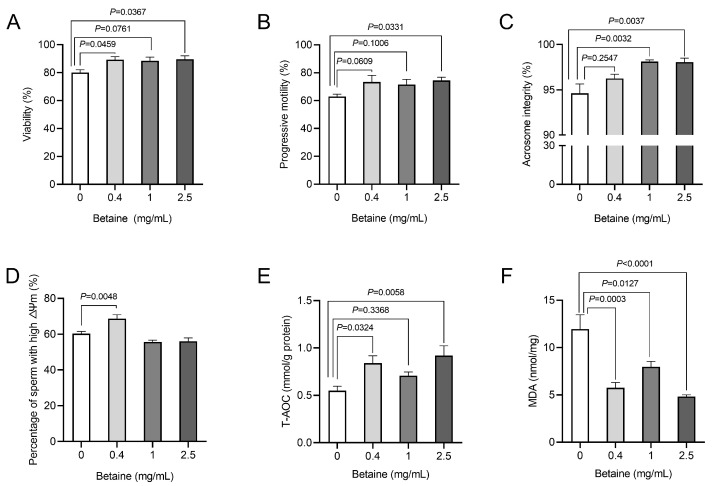
Betaine supplementation alleviated transport stress-induced damage of boar sperm. (**A**–**C**) Effects of betaine supplementation on sperm viability (**A**), progressive motility (**B**), and acrosomal integrity (**C**) during semen transport. (**D**) Effects of betaine supplementation on the percentage of sperm with high mitochondrial membrane potential. (**E**) Effects of betaine supplementation on sperm T-AOC content during semen transport. (**F**) Effects of betaine supplementation on sperm MDA content during semen transport. Values are expressed as mean ± SEM, n = 6.

The results indicate that betaine concentrations of 0.5 and 2.5 mg/mL exhibit superior protective effects. To delve deeper into its effects, we established concentrations of 0.4, 1, and 2.5 mg/mL within the range of 0–2.5 mg/mL in our actual road transportation model. All concentrations of betaine supplementation exhibited protective effects on sperm viability and motility during transportation. Notably, the 0.4 mg/mL and 2.5 mg/mL betaine supplementation groups exhibited higher sperm viability compared to the control groups (*p* = 0.0459; *p* = 0.0367, respectively, Figure 1A). The 2.5 mg/mL betaine supplementation group exhibited a significant increase in the percentage of sperm displaying progressive motility (*p* = 0.0331, Figure 1B). Furthermore, both the 1 mg/mL and 2.5 mg/mL betaine supplementation groups significantly enhanced sperm acrosomal integrity compared to the control group (*p* = 0.0032, *p* = 0.0037, respectively, Figure 1C).

Further examination of ΔΨm revealed enhanced mitochondrial function in sperm from the 0.4 mg/mL betaine treatment group (*p* = 0.0048, Figure 1D). Betaine supplementation could enhance the antioxidant capacity of boar sperm and mitigate lipid damage (Figure 1E,F). Supplementation with 0.4 and 2.5 mg/mL betaine significantly boosted the overall antioxidant capacity of sperm (*p* = 0.0324, *p* = 0.0058, respectively). Notably, all three concentrations greatly reduced lipid peroxidation damage (*p* = 0.0003, *p* = 0.0127, *p* < 0.0001, respectively).

### 3.3. Effects of Betaine Supplementation on Boar Sperm Quality during Simulated Temperature Fluctuations

**Figure 2:**
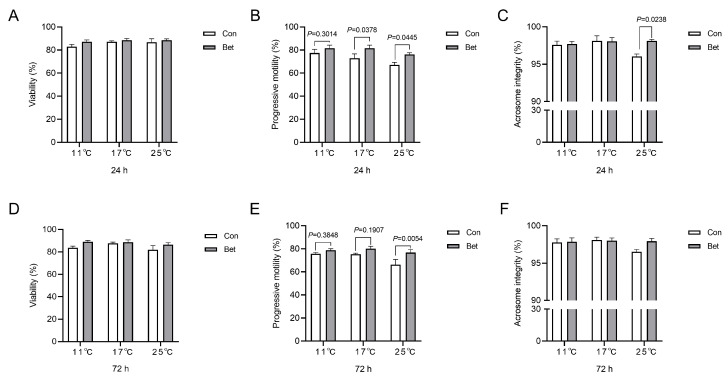
Boar sperm supplementation with betaine (0.4 mg/mL) under different temperatures (11 °C, 17 °C, and 25 °C) for 24 h and 72 h. (**A**–**C**) Effects of betaine supplementation on boar sperm viability (**A**), progressive motility (**B**), and acrosomal integrity (**C**) for 24 h. (**D**–**F**) Effects of betaine supplementation on boar sperm viability (**D**), progressive motility (**E**), and acrosomal integrity (**F**) for 72 h. Values are expressed as mean ± SEM, n = 6.

To assess the protective impact of betaine in boar semen extender for sperm against temperature fluctuations, we designed models to mimic temperature variations during transportation and identified a concentration of 0.4 mg/mL as being optimal. We observed no significant effects of betaine supplementation on boar sperm viability across various storage durations or temperature fluctuations (Figure 2A,D). At concentrations of 0.4 mg/mL, betaine supplementation did enhance the percentage of sperm with progressive motility when stored at both 17 °C and 25 °C for 24 h (*p* = 0.0378, *p* = 0.0445, respectively, Figure 2B). The most significant impact was observed in the sperm samples stored at 25 °C for 72 h (*p* = 0.0054, Figure 2E). Compared to the control group, betaine supplementation increased in the proportion of sperm with intact acrosomes when stored at 25 °C for 24 h (*p* = 0.0238, Figure 2C) but not 72 h (Figure 2F).

### 3.4. Effects of Betaine Supplementation on Boar Sperm Quality during Simulated Transportation Bumps

**Figure 3:**
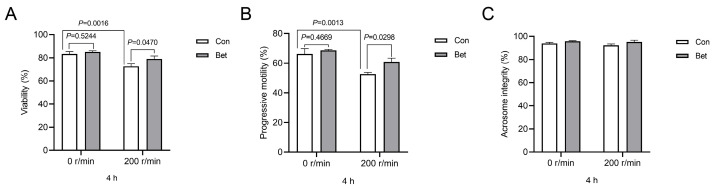
Betaine supplementation alleviated vibration emission-induced damage of boar sperm. Boar sperm supplementation with or without betaine (0.4 mg/mL) with vibration stress (0, 200 rpm/min) at 17 °C for 4 h. (**A**) Effects of betaine supplementation on boar sperm viability. (**B**) Effects of betaine supplementation on boar sperm progressive motility. (**C**) Effects of betaine supplementation on boar sperm acrosomal integrity. Values are expressed as mean ± SEM, n = 6.

To assess the protective effect of betaine at a concentration of 0.4 mg/mL against the detrimental effects caused by vibration exposure, we set vibration speed at 200 r/min to mimic transit bumps. As expected, exposure to vibration stress for 4 h significantly reduced boar sperm viability and progressive motility (*p* = 0.0016, *p* = 0.0013, respectively, Figure 3A,B). Although betaine supplementation did not significantly improve sperm viability and progressive motility in a calm state, it was effective in countering the damage caused by vibration at a speed of 200 r/min (*p* = 0.0470, *p* = 0.0298, respectively, Figure 3A,B). However, no significant difference was observed in acrosomal integrity between the betaine supplementation group and the control group under the stimulated speeds tested (Figure 3C).

## 4. Discussion

The pig industry worldwide has widely embraced AI due to its numerous benefits in enhancing reproductive efficiency and mitigating disease transmission, leading to significant economic gains [6]. Liquid form has been the primary method for collecting and extending fresh boar semen for commercial AI over the decades. Extenders are used to preserve sperm membrane stability, which is crucial for their viability [24,25]. During the AI process, diluted fresh boar semen is cooled to 17 °C before being transported to the sow farms from the boar studs, all of which is stressful to the sperm. Therefore, how to reduce the impaired fertilization ability of sperm caused by stress during this pre-AI process is a matter of great concern to practitioners. Among them, it is feasible and promising to add some protective substances to the extender. Betaine, as a common feed additive, is widely present in plants and animals and can be involved in the metabolism of organisms as a methyl donor [26]. While the protective effect of betaine on spermatozoa of different species has been noted previously, it has mostly been studied for its cryoprotective properties [16,27,28]. A study has shown that the addition of 102 mM betaine to the semen extender can improve sperm motility parameters [18] and reduce the adverse effects of dilution on sperm quality in pigs. However, it is not clear whether it can protect boar sperm during storage at room temperature (17 °C) and transportation.

Boar semen is typically stored at 17 °C from collection until the AI operation. The quality of semen deteriorates over time [29]. In this study, a model of boar semen preserved at room temperature was first established. The results showed that betaine supplementation at 0.5 mg/mL improved the total and progressive motility of boar sperm compared with the control group on days 3 and 5 of preservation. This is consistent with the findings of Lugar et al. [18]. It is noteworthy that this effect was not enhanced with increasing doses. The high concentration of betaine addition (12.5 mg) impaired motility, while the addition of 205 mM betaine in that study increased the proportion of sperm with abnormal tail, perhaps explaining the decrease in motility.

AI offers the advantage of reducing geographical restrictions, enabling access to high-quality boar semen and genetic material in diverse regions. However, the centralization of semen production facilities has increased long-distance transportation. Statistics from Brazil, Germany, and the United States show that the average distance for overland transport of boar semen is 5–1500 km and can take up to 12 h [20]. The temperature fluctuations during transportation and the unavoidable effects of road conditions on semen quality remain concerns. It has been shown that sperm quality decreased with increasing vibration intensity and transportation time and that longer storage time enhanced this effect [20]. In this study, an actual road transportation model was conducted. The results indicated that sperm viability, motility, and acrosome integrity, which are key indicators of fertilization potential, were significantly higher in the 2.5 mg/mL betaine-supplemented group compared to the control group. It suggests that betaine may alleviate the negative impact of road transport on sperm fertilization capacity.

Boar sperm is highly susceptible to reactive oxygen species (ROS) attack due to its plasma membrane rich in polyunsaturated fatty acids (PUFAs) [30]. Our previous studies have revealed that motile boar sperm exhibit elevated mitochondrial activity and higher levels of ROS compared to less motile sperm [31]. However, physical damage, such as concussion and rotation, will significantly increase the ROS produced by semen, causing peroxidative damage to the sperm plasma membrane structure and fluidity, and this effect will be even more serious than the case of spontaneous peroxidation [32]. The addition of antioxidants derived from plant extracts to sperm extenders has been shown to mitigate the adverse effects of storage on sperm quality [33]. Among them, the role of betaine in enhancing sperm antioxidant ability has been mentioned in previous studies [14,15,34]. Betaine supplementation at 0.4 and 2.5 mg/mL increased the total antioxidant capacity and decreased MDA—the main product of lipid peroxidation—levels in sperm. Our further studies showed that 0.4 mg/mL betaine significantly increased mitochondrial membrane potential and protected mitochondrial activity in boar sperm. Mitochondrial status determines the availability of ATP, the energy currency of the cell, which has a decisive effect on sperm motility and fertilizing ability [35]. The results above suggest that betaine supplementation may play a protective role by improving the antioxidant capacity of sperm and alleviating the transport stress injury during the actual transport.

Temperature during transport was tracked and correlated to sperm motility. Although the common operating procedure is to keep boar semen at 17 °C, studies have shown that during transport, the temperature may be higher than the final storage temperature if semen is transported within a few hours after collection and processing [11]. In the present study, a betaine supplementation of 0.4 mg/mL at 25 °C showed improvements in sperm viability, motility, and acrosome integrity. It has been claimed that motility-induced cellular stress may have a cumulative effect on temperature stress when boar sperm are cooled below 12 °C, the critical temperature at which cold shock becomes apparent [36]. Thus, our results suggest that 0.4 mg/mL betaine could be the ideal concentration to resist damage to sperm quality from temperature fluctuations during transport.

Vibration exposure during the transportation of liquid semen has been identified as a significant contributor to sperm damage [11]. The heightened sensitivity of freshly processed semen to vibration may be attributed to the combined effects of hydrodynamic forces on the sperm surface resulting from dilution stress immediately prior to vibration. The extent of cell damage is contingent upon the magnitude and duration of the vibration stress [37]. Additionally, the formation and subsequent rupture of air bubbles due to the agitation of semen during transportation have also been implicated as potential causes of sperm damage [37]. In our current study, treatment at 200 rpm/min led to a significant reduction in the viability and motility of boar sperm, aligning with the findings of Paschoal et al. [11]. However, the addition of 0.4 mg/mL betaine significantly mitigated this damage.

The results highlight the potential of betaine as an extender additive for safeguarding sperm fertility during liquid semen transportation. These findings provide valuable insights for optimizing semen handling and transportation protocols in AI practices.

## 5. Conclusions

In conclusion, the addition of an optimal concentration of betaine to the boar semen extender proves an effective strategy for mitigating the detrimental effects of storage and transportation on sperm quality during the AI process. The observed enhancement in sperm antioxidant capacity suggests a potential underlying mechanism for its beneficial effects, warranting further investigation. These findings provide a theoretical foundation for the utilization of betaine in pig production and AI procedures.

## Figures and Tables

**Table 1:** Compositions of boar semen extender.

Composition	Added Amount
D-glucose (g/L)	27.56
Trisodium citrate (g/L)	7.85
EDTA-2Na (g/L)	2.12
Sodium bicarbonate (g/L)	1
Tris (g/L)	5.56
Citric acid (g/L)	2.9
Penicillin-Streptomycin solution (100×)	10

**Table 2:** Effects of betaine supplementation on boar sperm total and progressive motility (%) stored at room temperature (17 °C).

Storage Time	Sperm Motility (%)	Betaine Supplementation (mg/mL)			
		0	0.5	2.5	12.5
Day 0	Total motility	94.76 ± 0.93			
	Progressive motility	89.11 ± 1.94			
Day 1	Total motility	89.43 ± 2.34 ^a^	91.23 ± 1.41 ^a^	88.49 ± 1.71 ^a^	38.61 ± 4.21 ^b^
	Progressive motility	80.81 ± 3.99 ^a^	83.18 ± 2.76 ^a^	79.73 ± 2.86 ^a^	20.24 ± 3.03 ^b^
Day 3	Total motility	86.86 ± 1.41 ^b^	90.49 ± 1.5 ^a^	90.11 ± 1.56 ^a^	30.64 ± 3.56 ^c^
	Progressive motility	77.13 ± 2.44 ^b^	81.23 ± 3.03 ^a^	81.18 ± 2.62 ^a^	13.69 ± 2.86 ^c^
Day 5	Total motility	84.03 ± 2.34 ^b^	87.78 ± 1.39 ^a^	85.3 ± 0.78 ^ab^	25.44 ± 2.98 ^c^
	Progressive motility	73.09 ± 3.1 ^b^	78.49 ± 1.79 ^a^	74.62 ± 1.12 ^b^	9.4 ± 2.15 ^c^

Values are expressed as mean ± SEM, n = 6. The different lowercase letters in the same row indicate significant differences (*p* < 0.05).

## Data Availability

All relevant data are within the manuscript.
